# Home Safe and Smart: A Pilot Program Integrating Smart Technology and Home Modifications for Aging in Place

**DOI:** 10.1093/geroni/igaf122.1883

**Published:** 2025-12-31

**Authors:** Jack Fried, Meredith Hughes, Portia Singh, Pamela Toto

**Affiliations:** University of Pittsburgh, Pittsburgh, Pennsylvania, United States; University of Pittsburgh, Pittsburgh, Pennsylvania, United States; University of Pittsburgh, Pittsburgh, Pennsylvania, United States; University of Pittsburgh, Pittsburgh, Pennsylvania, United States

## Abstract

Smart technology is an effective solution for improving person-environment fit and eliminating barriers to aging in place. Adding smart technology to traditional home modifications can be cost-effective and provide powerful solutions for creating a healthy home. Home Safe and Smart (HSS) is an evidence-informed program delivered by an occupational therapist and a handy person integrating low-cost smart technologies and home modifications to reduce disability, increase safety and promote aging in place for community-dwelling older adults.

**Objective:**

Describe procedures and preliminary results of a pilot program delivering Home Safe and Smart (HSS) in partnership with a regional health plan.

**Methods:**

HSS was developed and piloted (N = 100) with nursing facility (NF) clinically eligible and NF ineligible persons 60+ invited by their health plan. The in-home assessment included standardized measures of self-report and observed performance of activities of daily living (ADL), fear of falling, readiness for change and environmental assessment to set personalized goals for function and health. HSS participants were eligible to receive select smart technology, home modifications and up to 5 in-home visits for associated training.

**Results:**

Participants demonstrated 89% ADL improvement, 91% increase in readiness for change and clinically meaningful reduction in fear of falling. Average cost for smart technology and home modifications was $567 per participant. Most common smart technology provided include Amazon Echo Show, smart lighting, smart health devices and smart doorbells.

**Conclusion:**

Preliminary outcomes indicate HSS and smart technology can be an acceptable and feasible prevention program for aging in place offered through a regional health plan.

